# Investigation of Cerebral Hemodynamics During Endovascular Aspiration: Development of an Experimental and Numerical Setup

**DOI:** 10.1007/s13239-023-00660-8

**Published:** 2023-02-22

**Authors:** C. A. Luisi, A. Amiri, M. Büsen, T. Sichermann, O. Nikoubashman, M. Wiesmann, U. Steinseifer, M. Müller, M. Neidlin

**Affiliations:** 1 Department of Cardiovascular Engineering, Institute of Applied Medical Engineering, Medical Faculty, RWTH Aachen University, Pauwelsstr. 20, 52074 Aachen, Germany; 2 Clinic for Diagnostic and Interventional Neuroradiology, University Hospital Aachen, Pauwelsstr. 30, 52074 Aachen, Germany

**Keywords:** Cerebral artery model, Cerebral blood flow, Particle image velocimetry, Computational fluid dynamics, Aspiration thrombectomy

## Abstract

**Purpose:**

Acute ischemic stroke is a life-threatening emergency caused by an occlusion of a cerebral artery through a blood clot. Aspiration thrombectomy is an endovascular therapy for the removal of vessel occlusions. However, open questions regarding the hemodynamics during the intervention remain, motivating investigations of blood flow within cerebral arteries. In this study, we present a combined experimental and numerical approach to analyze hemodynamics during endovascular aspiration.

**Methods:**

We have developed an *in vitro* setup for investigations of hemodynamic changes during endovascular aspiration within a compliant model of patient-specific cerebral arteries. Pressures, flows, and locally resolved velocities were obtained. In addition, we established a computational fluid dynamics (CFD) model and compared the simulations during physiological conditions and in two aspiration scenarios with different occlusions.

**Results:**

Flow redistribution within cerebral arteries after ischemic stroke is strongly dependent on the severity of the occlusion and on the volume flow extracted by endovascular aspiration. Numerical simulations exhibit an excellent correlation of *R* = 0.92 for flow rates and a good correlation of *R* = 0.73 for pressures. Further on, the local velocity field inside the basilar artery had a good agreement between CFD model and particle image velocimetry (PIV) data.

**Conclusion:**

The presented setup allows for *in vitro* investigations of artery occlusions and endovascular aspiration techniques on arbitrary patient-specific cerebrovascular anatomies. The *in silico* model provides consistent predictions of flows and pressures in several aspiration scenarios.

**Supplementary Information:**

The online version contains supplementary material available at 10.1007/s13239-023-00660-8.

## Introduction

In the past, ischemic stroke treatment was limited to intravenous thrombolysis (IV) with pharmaceuticals within a time window of 4.5 h after the occurrence of a cerebral artery occlusion.^7^ Since the first endovascular thrombectomy (EVT) device for mechanical clot removal was introduced in 2004, EVT evolved and has become a valid therapy for patients affected by acute ischemic stroke within a time window of up to 24 h.^8,12^ Now, stent retriever thrombectomy (SRT) and aspiration thrombectomy (AT) are the most common endovascular treatment strategies for large vessel occlusions.^2,11,25^ An important factor for procedural success is the knowledge of the hemodynamics within the Circle of Willis (CoW) anatomy. This includes information on blood flow rates and directions within the CoW during the intervention, especially considering the anatomy of the collateral vessels. For instance, intact anterior and posterior communicating arteries lead to more favorable EVT outcomes.^30^ However, information on the patient-specific hemodynamics during acute ischemic stroke is still very limited.

So far, various groups have measured blood flow *in vivo* within different cerebral arteries in healthy humans. Zarrinkoob *et al*. determined total cerebral blood flow (tCBF) in a study population of 94 subjects via phase contrast magnetic resonance imaging (PC-MRI).^29^ Van Ooji *et al*. visualized flow within the CoW with PC-MRI, finding that flows through the communicating arteries, presumed to be responsible for flow redistribution within the CoW, were unilateral over the cardiac cycle.^26^ Correia de Verdier and Wikström measured flow and velocity parameters in six intracranial cerebral arteries of 30 healthy young (18 to 49 years) subjects *via* PC-MRI.^3^

Fahy *et al*. performed *in vitro* studies during a simulated cardiac cycle on three patient-specific cerebral artery models with a complete CoW using six ball and needle valves to adjust the efferent vessel flows and compliance chambers to keep the pressure of the efferent vessels in a physiological range.^6^ In a follow-up study, the pressure and flow redistribution in efferent cerebral arteries after occlusion of different artery segments in one model was investigated. The authors showed that clotting an MCA segment shunts the flow towards the contralateral side.^5^ However, flow fields inside the model and flow within the communicating arteries were not measured.

Different research groups performed numerical analyses in order to gain better understanding of cerebral hemodynamics in complete and incomplete CoW models.^1,10,17^ Luraghi *et al*. implemented a computational model of mechanical thrombectomy with a stent retriever.^14^ Other researchers focused on the effects of aspiration thrombectomy for ischemic stroke modelling a cylindrical vessel segment.^13,20^ Neidlin *et al*. performed a numerical investigation on hemodynamics in a full CoW during distal thrombus aspiration.^18^

In the studies above, experimental and numerical investigations of thrombectomy have been performed separately. In the extensive review by Luraghi *et al*.^15^ on models for EVT, it is mentioned that there exist only two studies in the literature that use a combined *in vitro* and *in silico* approach. However, the first study^16^ models thrombectomy with a mechanical stent retriever and does not look at blood flow in the CoW and its changes during aspiration. The second study only looks at aspiration in a cylindrical segment and disregards the complex CoW anatomy.^9^

Thus, our study aims to present a combined experimental and numerical setup for the analysis of cerebral hemodynamics during endovascular aspiration in a realistic vascular model. On the experimental front, it addresses the following four aspects—(a) model a patient-specific vascular anatomy, (b) deliver flows, pressures and pulsatility indices like the ones observed *in vivo*, (c) allow vessel obstruction and use of aspiration catheters, and (d) measure flows and pressures and perform spatially resolved flow visualization. On the numerical front, an initial computational fluid dynamics (CFD) model for the calculation of flows and pressures and its comparison to experimental data is presented.

## Materials and Methods

### Experimental Setup

**Figure 1:**
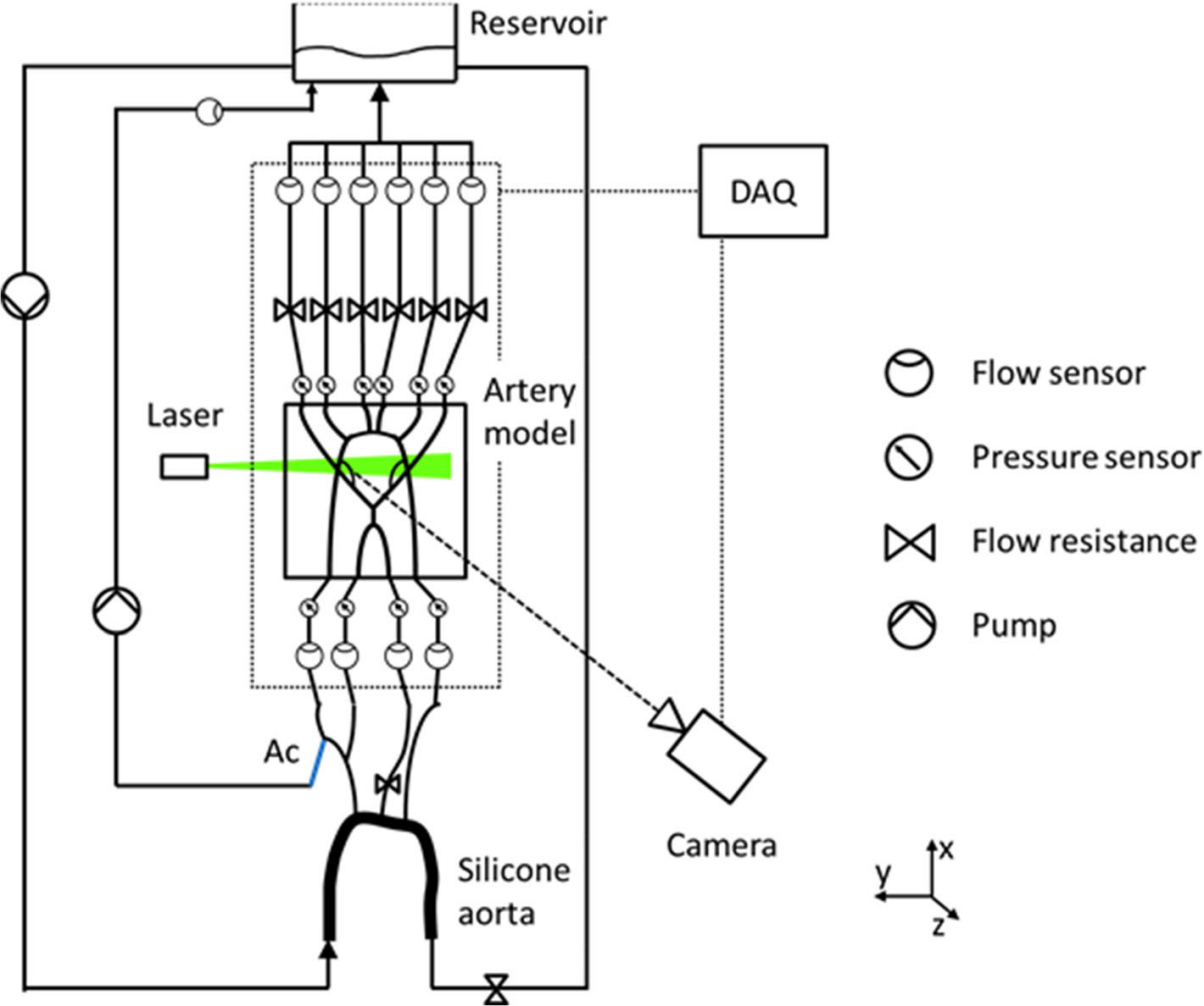
Scheme of the experimental setup. *Ac* aspiration catheter, *DAQ* data acquisition.

An experimental setup has been developed allowing *in vitro* flow studies in a model of cerebral arteries by occluding specific arteries and simulating endovascular aspiration. Detailed flow information was generated with PIV as the flow measurement technique providing velocity fields. During experiments, flows and static pressures of afferent and efferent vessels were monitored simultaneously. The experimental setup is shown in Figure 1.

Pulsatile flows of a water–glycerol mixture were generated through a 60 mL displacement pump (Driving System, Medos, Germany) at a nominal heart rate of 60 heartbeats per minute. A compliant silicone model of the aortic arch was placed after the pump in order to mimic the effect of the natural vascular compliance on the pressure curves. In this manner, physiological pressure curves at the inlets of the artery model were generated. The water-glycerol mixture had a density of 1.095 ± 0.02 g/mL and a viscosity of 3.51 ± 0.07 mPa s. The mixture had a mass fraction of 40% glycerol without further additives. The cerebral artery model was embedded into an acrylic glass compartment with 210 × 145 × 90 mm^3^ inner dimensions. The glass compartment was filled with the same water-glycerol mixture. Connectors were sealed for holding the silicone model inside the compartment and allowing connection of the pressure sensors and outlet tubes outside of the acrylic glass compartment. Outlet tubes downstream of the model were quenched with adjusting screws in order to represent the total resistance of the capillary bed. The total cerebral flow was regulated by the bypass-flow resistance downstream of the aortic arch.

### Cerebral Artery Model

A flexible and hollow silicon cerebral artery model was designed and manufactured in six steps.

The cerebral arteries of a 74-year-old female stroke patient with a complete CoW were segmented from an anonymized CTA-scan. The data handling was approved by the ethics review committee of the University Hospital Aachen. Centerlines were extracted from the segmented data using 3-matic and Mimics software (Materialise, Leuven, Belgium), giving a patient-specific curvature of cerebral arteries. In the second step, vessel diameters were derived from an existing CFD model^18^ and imposed through sweeping operations on the obtained centerlines. The outflow branches were extended to the connectors of the acrylic compartment in order to allow a modular exchange. Four afferent arteries defined the inflow: left and right internal carotid arteries (LICA and RICA) and left and right vertebral arteries (LVA and RVA). Six efferent vessels defined the outflow: left and right posterior cerebral arteries (LPCA and RPCA), left and right middle cerebral arteries (LMCA and RMCA) and left and right anterior cerebral arteries (LACA and RACA). The resulting dimensions of the vessel network and the names of the arteries are shown in Supplementary Table 1.

**Figure 2:**
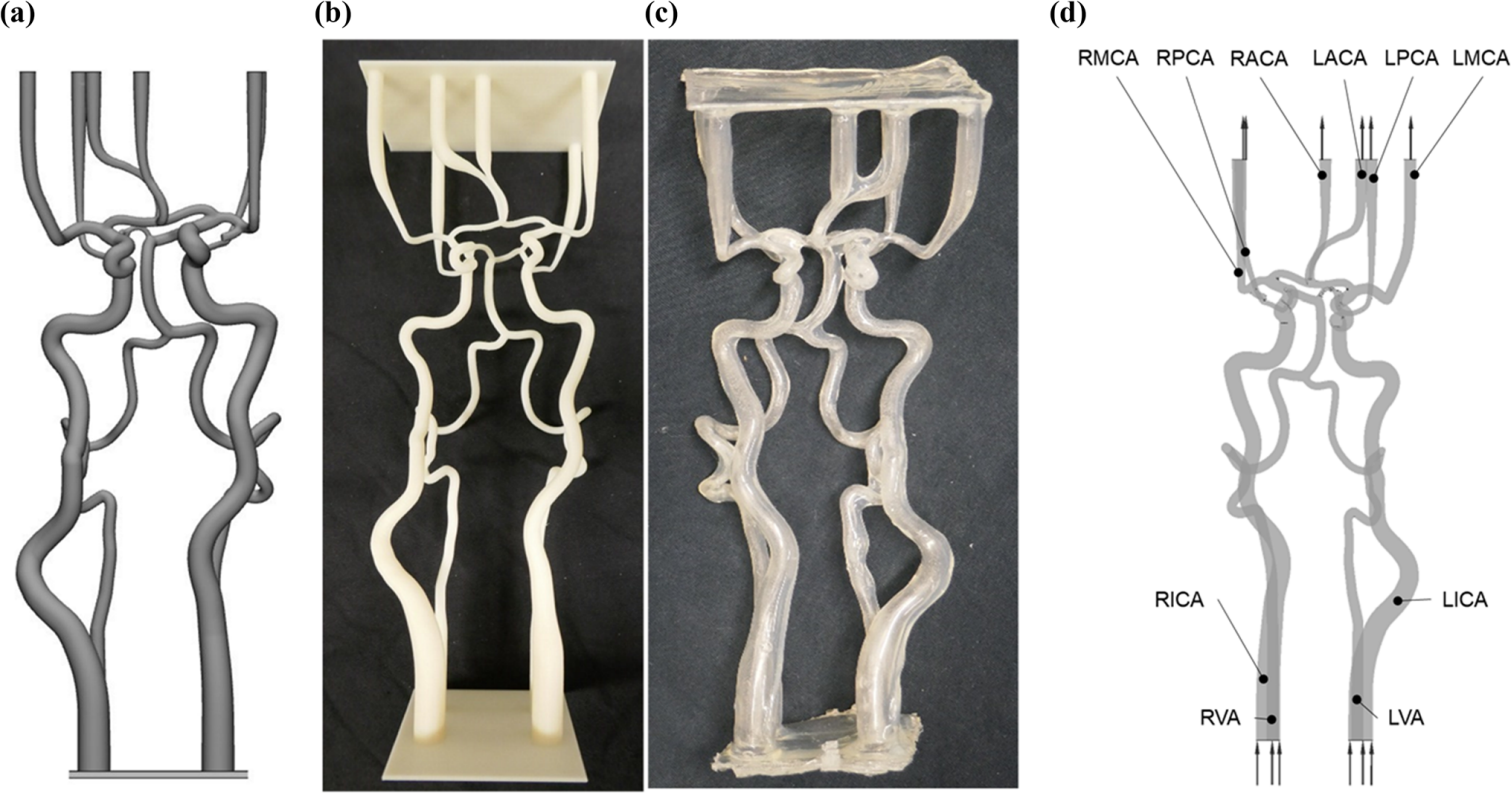
Example of the process from designed to manufactured silicone model. (a) CTA-data based segmented model with adaptation at inlet and outlet, (b) printed ABS core, (c) hollow, flexible silicone model (d) CFD model of the CoW with inlets and outlets.

Afterwards, the core-geometry was printed with the thermoplastic polymer ABS via an industrial 3D printer (FORTUS 450mc, Stratasys, Eden Prairie, USA) with a layer thickness of 0.127 mm. The fourth step consisted in optically smoothing the surface of the ABS core-geometry in acetone-saturated air at 23 °C in a closed box for 50 min before curing one day at laboratory conditions (23 °C, ambient pressure). Then the core was thoroughly dipped in a two-component silicone rubber (RT 625, Wacker Chemie AG, Köln, Germany). Uniaxial tension tests of the silicone rubber for small strains < 0.2 yielded nearly linear elastic material behavior with a Young’s modulus of approximately 0.53 MPa. Immediately after dipping, the model was rotated simultaneously in three spatial axes until the vulcanization process had progressed so that the silicone became stable. Finally, the cured silicone model of cerebral arteries was rinsed in acetone for 24 h so that the ABS completely dissolved, leaving a flexible, hollow silicon model. The results of the model manufacturing steps are shown in Figs. 2a–c.

### Measurement Instrumentation

All inflows to the silicone artery model and its outflows were measured with ultrasound flow sensors (Transonic Systems Inc., Ithaca, USA) at 0.15 m distance from the compartment and the same distance to the silicone aortic arch (see Fig. 1). Analog signals were captured with a T470 Flowmeter (Transonic Systems Inc., Ithaca, USA). Static pressures were measured with pressure transducers (Xtrans, CODAN pvb Medical GmbH, Lensahn, Germany) for all inflows and outflows at the connector positions and the mean arterial pressure (MAP) was measured at the inlet of the silicone aortic arch. All flow and pressure signals were acquired simultaneously at 100 Hz (Labview, National Instruments, Austin, USA). In order to determine flow velocities inside the silicone model, PIV measurements were performed with a double pulse Nd:YAG laser source (EverGreen70, Quantel, Les Ulis, France) at a wavelength of *λ* = 532 nm. Rhodamine B coated polystyrene particles with a diameter of 10.5 ± 2 *µ*m and a density of 1.06 g/mL (ILA GmbH, Gernsheim, Germany) were used to seed the fluid. The light-scattering particles were distinguished from noise signal by applying a 570 nm long-pass filter during image acquisition.

**Figure 3:**
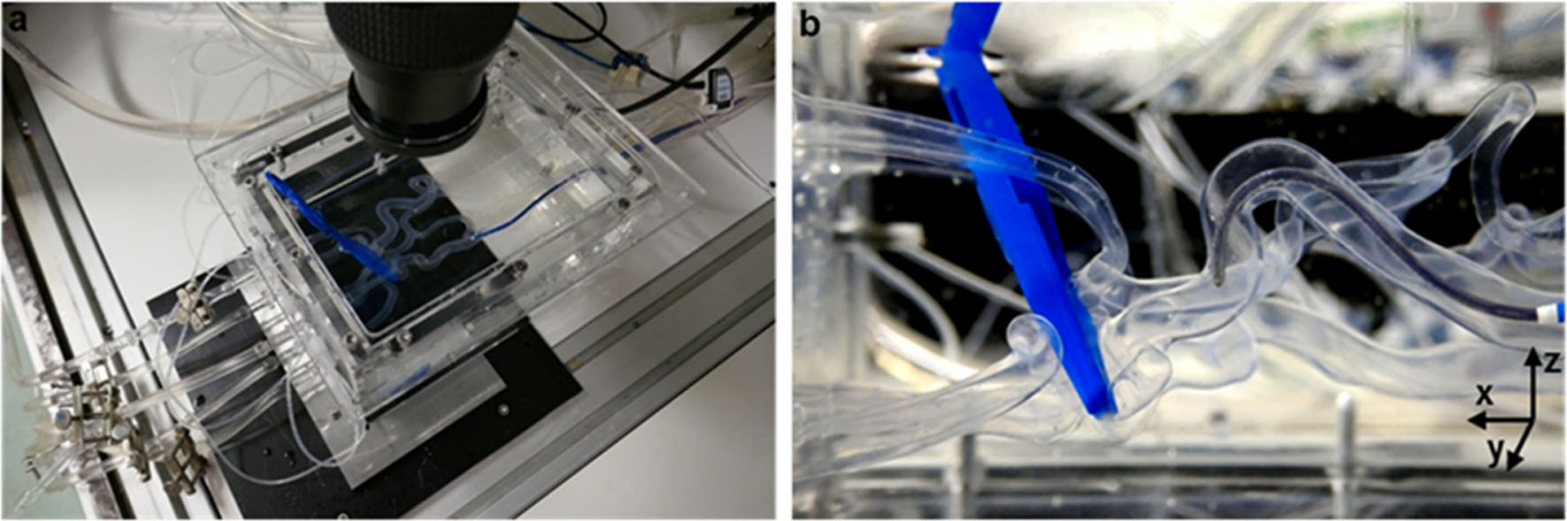
(a) Image of the experimental setup with cerebral artery model. (b) Vessel occlusion and aspiration catheter.

Image acquisition was performed with a 1024 × 1280 Pixel CMOS sensor (NanoSense MK3, Dantec Dynamics, Skovlunde, Denmark). In every measurement plane a set of 450 images was acquired with a frequency of 15 Hz, corresponding to 30 pump cycles. A resolution of 0.016 mm/pixel was achieved with the used lens (Micro-Nikkor 60 mm f/2.8D, Nikon, Tokio, Japan). Cross-correlation algorithms and further post-processing of the acquired images were performed with the software package DynamicStudio 6.10.67 (Dantec Dynamics, Skovlunde, Denmark). The presented vector maps were averaged over the pump cycles and the final vector spacing was 0.256 mm. The photograph of the experimental setup is shown in Fig. 3a.

### Measurements

At total, four scenarios A-D were investigated. In the physiological scenario, scenario A, flow resistances were adapted in order to obtain flow rates corresponding to *in vivo* measurements in healthy subjects.^28^ Flow resistances were not changed during the experiments and the pulsatility indices for the vessel segments were then determined by the vascular model and the pulsatility of the flow through the aortic arch. Values for the pulsatility indices were derived from flow measurements *Q*_*i*_ according to Eq. 1.

1 $${\text{PI = }}\frac{{{ }Q_{{{\text{systole}}}} { } - { }Q_{{{\text{diastole}}}} }}{{Q_{{{\text{mean}}}} }}$$

Three exemplary cycles of the acquired flow curves for LICA and RICA are shown in Supplementary Fig. 1 to illustrate the pulsatility of the flow and repeatability of the experimental measurements. The raw data and the STL model can be found here 10.5281/zenodo.6958474.

**Figure 4:**
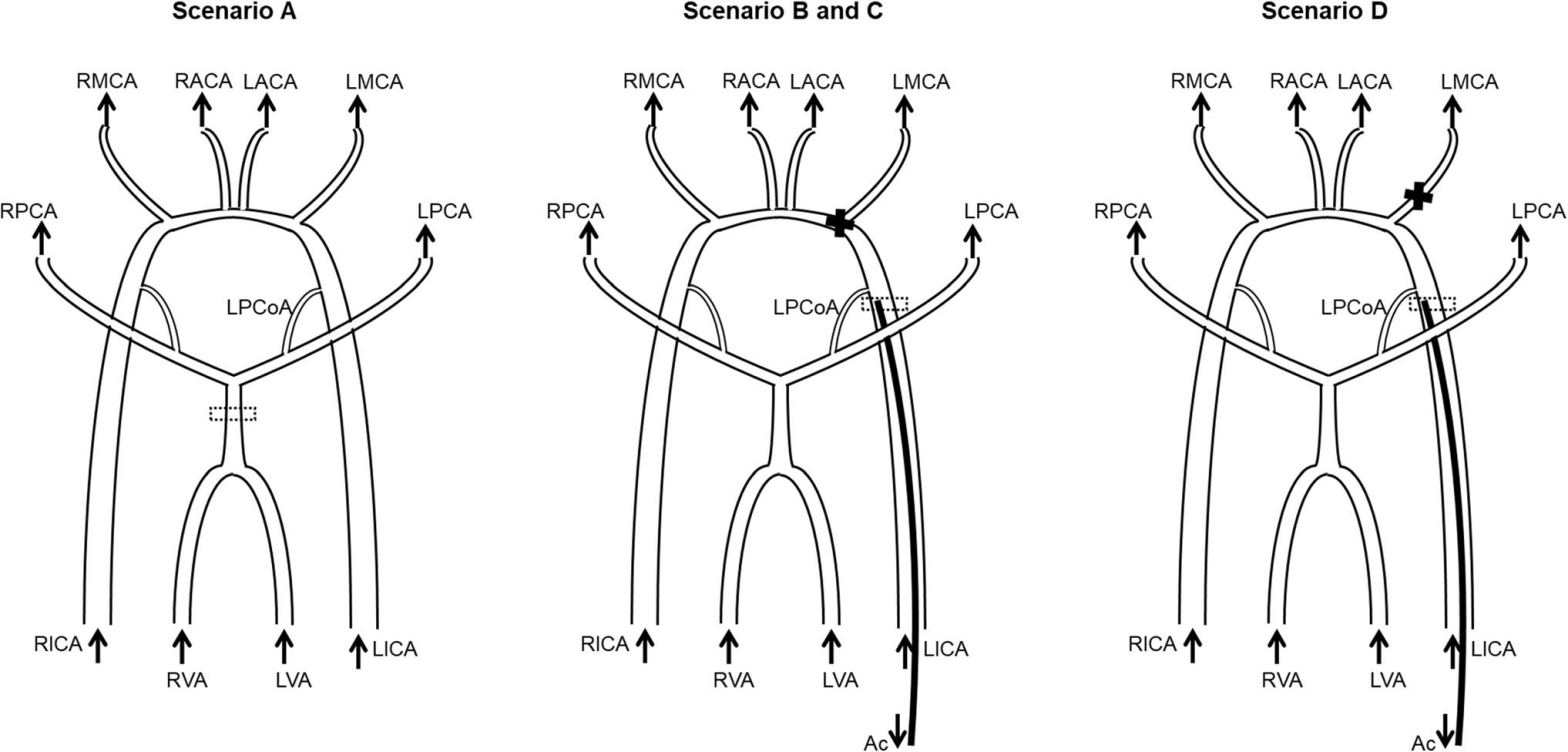
Schematic view of flow scenarios. Thrombus visualized by black cross. PIV measurement windows shown by dotted frame. (a) physiological scenario. (b) and (c) A T-occlusion with aspiration catheter. (b)—no aspiration, (c)—aspiration of 100 mL/min. (d) An LMCA occlusion with aspiration catheter and an aspiration of 100 mL/min.

In the remaining flow scenarios, the influence of an aspiration catheter and endovascular aspiration during large vessel occlusions was investigated. The aspiration catheter had a 1.524 mm inner diameter (1.8 mm outer diameter) (Catalyst 6, Stryker, USA) and was placed inside the LICA, proximal to the LPCoA junction, as seen in Fig. 4. The generated large vessel occlusions were *T-occlusion* (occlusion of the terminal carotid artery as well as the anterior and middle cerebral artery) and *LMCA occlusion*,^2^ an occlusion inside the LMCA, 2 mm downstream of the junction, respectively. Occlusions were generated by clamping of the silicone model, see Fig. 3b.

The influence of the positioning of an aspiration catheter was analyzed within scenario B, where no flow was extracted through the catheter during experiments with a *T-occlusion*. In scenario C, with the same *T-occlusion*, a peristaltic pump (323 SciQ, Watson-Marlow GmbH, Rommerskirchen, Germany) extracted 100 mL/min through the aspiration catheter, a value based on^20^ and preliminary experiments. The same setting was analyzed for an *LMCA occlusion* in scenario D.

For the pathological scenarios, inflow at the LICA could not be measured with ultrasound flow sensors, as the catheter interfered with the ultrasound signal. For these cases, flow inside the LICA was determined by evaluating image sets acquired with PIV around the catheter tip. The velocity vector fields derived for each plane were averaged over the pump cycles. From a set of 7 to 10 planes, the shape of the velocity field was reconstructed with a visualization software (Tecplot 360 EX 2015, Bellevue, USA) and volume flow was determined by multiplying the mean velocity in x-direction with the vessel area in the *y*–*z* plane. We validated this approach by evaluating the mass balance during scenario B in the basilar artery (BA) and identified an error of < 1% between the volume flow determined by PIV and by mass balance. A schematic view of the scenarios is shown in Fig. 4. All experiments were conducted at constant laboratory conditions at 23 °C and ambient pressure.

### Numerical Setup

The same geometry was used for CFD simulations with the vascular occlusions (scenarios B, C, and D) represented by introducing walls into the model. The geometry for every scenario was meshed with unstructured tetrahedral elements and five layers of prismatic elements in the boundary layer of the vessels and three layers in the boundary layer of the catheter. For the mesh independence study, the mass flow rates in the outlets were taken as the control parameters and the number of elements for the final mesh ranged within 1.9–2.4 × 10^6^ for four scenarios (A, B, C, and D). The CFD models were created with CFX 20.0 (ANSYS 20, Ansys Inc., Canonsburg, USA). Fluid was a water-glycerol mixture with a density of 1.095 g/mL and a dynamic viscosity of 3.51 mPa s. The simulation was steady state and the flow was classified as laminar because the Reynolds numbers at the vessel inlets and outlets were between 18 and 297. An RMS value of 10^–4^ was assumed for the convergence criterium.

For the boundary conditions, mass flow rates at the inlets and pressures at the outlets were set. The values were cycle averaged from the transient experimental data. In scenarios C and D, the catheter outlet was set with a constant volume flow rate of 100 mL/min. The CFD model is further shown in Fig. 2d.

## Results

We evaluated flow parameters and static pressures for a patient-specific cerebral artery model. Flow distributions and MAP were compared among the analyzed scenarios in order to gain information on the effects of different artery occlusions, positioning of the aspiration catheter, as well as performance of endovascular aspiration. In addition, a comparison to numerical simulations was performed.

### Physiological Scenario

Data obtained with the physiological scenario and the relevant literature values are presented in Table 1. Total flow is in accordance to *in vivo* data (657 ± 94 mL/min) for healthy elderly subjects.^29^ Mean flow rates for the outflow vessels (LPCA, RPCA, LMCA, RMCA, LACA, RACA) are in compliance to values derived from References ^3,26^. However, the inflow distribution is accentuated at the RICA in our setup and less pronounced at LVA, which has a higher PI then the reported literature value. Nevertheless, the PI values determined in this work, besides the one for the RPCA as pointed out in the comparison section, are within the range of literature values.^3,28^ MAP has a value of 98.5 mmHg and is in accordance to reported physiological values.^28^

**Table 1:** Mean flow rates and PI values for the physiological scenario A in this study and other *in vivo* literature values.

Vessel	This study		van Ooij et al.^26^	Correia de Verdier and Wikström^3^		Zarrinkoob *et al*.^28,29^	
	Mean flow rate ± SD [ml/min]	PI ± SD [–]	Flow rate ± SD [mL/min]	Flow rate (range) [mL/min]	PI (range) [–]	Flow rate ± SD [mL/min]	PI ± SD [–]
total	614 ± 24	–	636	697	–	657 ± 94	–
LICA	233 ± 13	1.04 ± 0.08	240 ± 42	–	–	236 ± 41	0.96 ± 0.15
RICA	292 ± 16	0.94 ± 0.07		–	–		
LVA	40 ± 4	1.86 ± 0.18	–	–	–	90 ± 17	1.11 ± 0.18
RVA	57 ± 4	1.20 ± 0.11	–	–	–		
LPCA	74 ± 3	0.74 ± 0.04	60 ± 18	77 (31–133)	0.58 (0.26–1.00)	51 ± 10	0.85 ± 0.13
RPCA	63 ± 3	0.90 ± 0.06		72 (22–115)	0.56 (0.16–0.86)		
LMCA	162 ± 6	0.61 ± 0.03	186 ± 48	169 (111–255)	0.71 (0.38–1.54)	131 ± 23	0.84 ± 0.13
RMCA	170 ± 6	0.60 ± 0.03		174 (127–264)	0.69 (0.44–1.64)		
LACA	69 ± 3	0.77 ± 0.04	72 ± 18	93 (28–195)	0.60 (0.18–1.57)	27 ± 8	0.77 ± 0.12
RACA	78 ± 3	0.69 ± 0.03		113 (36–190)	0.67 (0.23–1.19)		

### Pathological Scenarios

**Figure 5:**
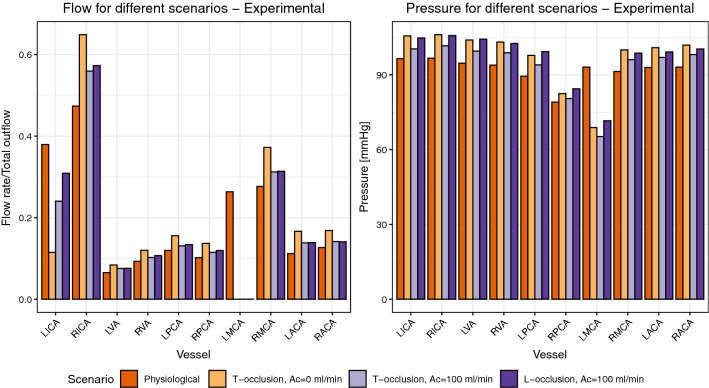
Flows and pressures for scenarios (a–d). The flow rates are normalized by the total outflow, (a)—614 mL/min, (b)—554 mL/min, (c)—616 mL/min and (d)—652 mL/min.

Pressure and volume flow data for scenarios B, C and D are presented in Fig. 5. Total outflow is defined as the sum of all outlet flows including the aspiration catheter (Ac) flow. For the sake of comparability between the analyzed scenarios, values for the reported flow rates are divided by the total outflow for each scenario. These total outflows are: scenario A—614 mL/min, scenario B—554 mL/min, scenario C—616 mL/min and scenario D—652 mL/min.

The deviation of sum of inlet flows to the sum of outlet flows and therefore the uncertainty in flow measurements is < 2%. The uncertainty of pressure values was estimated ± 2% during calibration experiments. In scenario B, a *T-occlusion* without flow extraction by the aspiration catheter, the total flow is 70 mL/min lower than in the physiological scenario. Furthermore, the vessel occlusion and insertion of the aspiration catheter shifts the blood flow of the LICA to the contralateral side, increasing the RICA flow. In general, flow rates of the right afferent vessels are higher than the left side as obtained from the experiments. Efferent vessels such as ACA and PCA have similar outflows on both sides of the model. However, a difference in flows between LPCA and RPCA is observable, which can be attributable to a lower diameter of the RPCA in our silicone model due to a stenosis that occurred during production and that was not noticed prior to our experiments. This is substantiated by an about 10 mmHg lower pressure for the RPCA when compared to the LPCA, see Fig. 5 on the right. Further, we reconstructed the stenosis in our CFD simulations from microscopic images and simulated scenario A. Indeed, the RPCA flow dropped from 144 to 101 mL/min, thus confirming our hypothesis of the stenosis being the reason for the differences, see Supplementary Fig. 3 and Supplementary Table 2. In scenario C, with the same occlusion as in B, flow is now extracted from the aspiration catheter in the LICA. This yields a flow reduction in all vessels except for the LICA. Once again, the flows on the left and right side are rather balanced and only the ICA flows exhibit large differences. In scenario D, an occlusion of the LMCA with aspiration, leads to an increased total flow. Compared to scenario C, having an additional flow path from LICA to LACA, flow distribution towards the RICA is less pronounced.

For scenarios B to D, the MAP and all vessel pressures, except for the occluded LMCA, are increased compared to the reference scenario A. The static pressure of the occluded vessel LMCA in turn is lower for scenarios B to D compared to scenario A.

### Comparison *In Silico *with *In Vitro*

**Figure 6:**
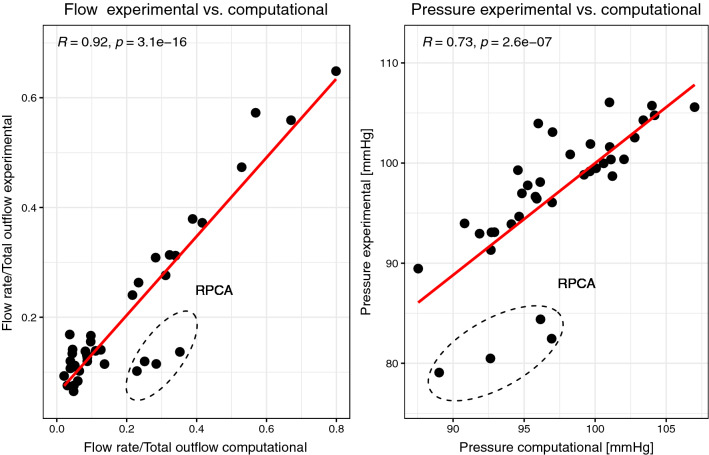
Scatterplots of flows and pressures for all scenarios. Comparison of computational and experimental data with Pearson correlation. The dashed ellipse shows the outliers belonging to RPCA. Red line from linear regression.

Figure 6 compares the experimental and computational results in all scenarios A-D at all vessel boundaries for the flow rates and the pressures, respectively. For the flow rates, an excellent correlation with Pearson’s *R* = 0.92, *p* < 0.001 is determined. The pressure values have a good correlation with *R* = 0.73, *p* < 0.001. A linear regression is shown by the red line. Outliers are observable for both, flows and pressures, which are marked by the dashed ellipse in Fig. 6 and are attributed to the RPCA. An individual comparison of pressures and flows between experiment and simulation is shown in Supplementary Fig. 2.

**Figure 7:**
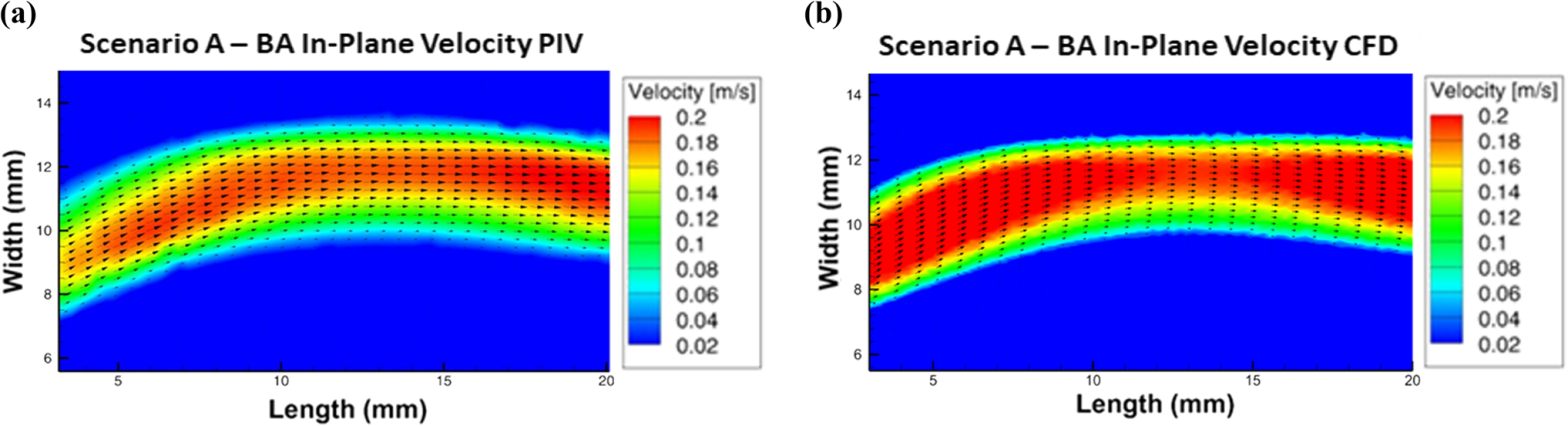
BA field in-plane velocities, (a) Scenario A acquired via PIV, (b) Scenario A calculated via CFD.

The velocity field in a plane located within the BA was compared for scenario A between PIV and CFD in Fig. 7. A comparison yields good correspondence between experiment and simulation considering the overall velocity distribution and the shape of the central flow. CFD exhibits larger regions of high velocities, especially at the boundaries of the cross-section.

## Discussion

In this work, we presented the development of a combined experimental and numerical setup for investigations of hemodynamics during endovascular aspiration. Physiological flow and two vessel occlusions (carotid *T-occlusion* and *LMCA occlusion*) with and without aspiration have been investigated. Volume flow and pressure curves were acquired simultaneously and detailed velocity fields inside specific areas of the model were captured with the PIV measurement technique. A comparison with *in silico* simulations has been carried out as well.

Flow distributions within the silicone artery model are consistent with physiological measurements *in vivo*.^3,6,26^ Pulsatility indices are widely in accordance to *in vivo* data as well.^3,28^ Our results for pathological scenarios qualitatively agree with prior findings^5^ regarding flow redistribution after vessel occlusion. However, no *in vivo* data investigating endovascular aspiration after ischemic stroke was available for validation.

The numerical model corresponds well with the experiments and, even considering the measurement errors in the RPCA caused by model flaws, yields an excellent correlation coefficient of *R* = 0.92 for the mass flows and *R* = 0.74 for the pressure values. The steady-state CFD simulations have a computational runtime of approximately 5 min, thus a wide variety of scenarios e.g. different catheter locations, aspiration flows or different geometries can be investigated within this numerical setup. During operation, an oscillation of the thin-walled model has been observed making a quantitative comparison between CFD and PIV extremely difficult. This oscillation was caused by pressure waves produced by the displacement pump. For a more detailed analysis, a casted silicone block with the negative of the CoW might be used, however this will most certainly weaken the model's capability of producing physiological PI values as they were achieved in this study.

Comparison between CFD and PIV on a plane located in the BA has shown good agreement regarding velocity distribution and shape of the velocity field.

The results give insights on how thrombectomy can be further improved considering the presented positioning of the aspiration catheter. During aspiration with a *T-occlusion*, the fluid acceleration into the catheter tip is small, suggesting that the induced flow force might be too low to effectively remove blood fragments from the chosen proximal position. In case of *LMCA occlusion*, the aspiration—as it was performed—had no effect on blood flow direction inside the ICA. In case of a blood fragment dissociated inside the MCA vessel, the fragment could be transported towards the efferent vessels causing further neurological damage. In order to enhance fragment aspiration, positioning of the catheter near to the occluded segment is an essential prerequisite, but also the occlusion of the ICA by inflating a balloon catheter seems to be required for this purpose.^21^ This is consistent with the retrospective radiological study by Schönfeld *et al*. who have shown that there are smaller and less distal emboli if balloon guide catheters are used.^23^ In addition, catheter tip proximity with the thrombus has been shown to be very important for a successful aspiration in experimental and numerical studies.^9,19,22,24^ There are several limitations to this work. First, for proving the measuring concept, only one simplified arterial model was developed; thus, no generalization of flow redistribution due to endovascular aspiration is possible at the moment. Second, the out-of-plane component of the velocity field was not captured with the present setup, leading to potential underestimation of flows mostly in curved vessels. Third, cerebral autoregulation, which maintains cerebral blood flow in response to a varying arterial pressure, was not simulated, leading to potential underestimation of mean total outflow *in vitro* and thus overestimation of PI-values. Several improvements of the experimental setup would increase the clinical implication of the work. At first, more tip locations with closer clot proximity should be investigated. Secondly, the use of balloon guided catheters, which allow flow arrest in the afferent arteries (e.g. internal carotid artery) are a highly clinically relevant scenario. Finally, use of a pressure-controlled aspiration pump instead of a flow-controlled peristaltic pump is advised, as the aspiration pumps are the ones that are used in clinical practice.

On the numerical side, more complex model setups are imaginable including transient simulations, fluid–structure interaction simulations and coupling of Windkessel elements at the model outlets. Besides increased numerical costs, model uncertainties especially regarding material parameters exist. However, coupling of a lumped parameter model to the 3D CFD setup, like it has been performed in other cardiovascular applications^4,27^ might be an interesting option to explore. Such multiscale models can be used to obtain better insight of the system response towards aspiration and allow for more realistic boundary conditions. At last, a pressure boundary condition at the cannula tip (instead of the flow rate boundary condition) would be a much more realistic scenario if one wants to simulate the flow during the contact of the catheter with the clot.

Despite these limitations, our setup already provides detailed insight into fluid mechanics inside the CoW. As *in vivo* measurements are hardly feasible during ischemic stroke and thrombectomy, *in vitro* studies offer basic understanding of the underlying mechanisms in cerebral arteries. The capabilities of simultaneous acquisition of time-resolved pressures and flows opens up many opportunities to further investigate still largely unknown factors of aspiration thrombectomy. Examples include the influence of vessel stiffness on CoW blood flow or other aspiration modes during the intervention (pulsatile instead of constant).

On top of this, *in silico* models provide the opportunity to evaluate a large parameter space and compare various intervention scenarios with each other. A possible requirement to improve clinical treatments with EVT for patients with acute ischemic stroke is to understand hemodynamic effects in cerebral arteries during such interventions. For this purpose, future work needs to address the influence of patient-specific geometries of cerebral arteries on physiological hemodynamics as well as during interventional procedures.

## Conclusion

In this study, we presented a combined experimental and numerical approach that can help to understand hemodynamic effects in cerebral arteries not only under physiological conditions, but most importantly in case of ischemic stroke and especially during endovascular aspiration.

The *in vitro* setup uses a patient-specific anatomy and reproduces physiological flow rates and pulsatilities observed *in vivo*. Experimental flow visualization in larger arteries of the silicone model is feasible with PIV and flow during vessel occlusions and endovascular aspiration scenarios can be analyzed. The *in silico* model provides consistent predictions of flows and pressures considering time-averaged quantities.

## Supplementary Information

Below is the link to the electronic supplementary material.

Supplementary file1 (DOCX 1810 KB)
