# Supplementary material for: Investigation of Cerebral Hemodynamics During Endovascular Aspiration: Development of an Experimental and Numerical Setup
Source: Cardiovasc Eng Technol. 2023 Feb 22;14(3):393–403. doi: 10.1007/s13239-023-00660-8 (PMC10412675; doi:10.1007/s13239-023-00660-8)
Supplement: Supplementary file 1 — Supplementary file1 (DOCX 1810 KB) [file 13239_2023_660_MOESM1_ESM.docx]

**Investigation of Cerebral Hemodynamics during Endovascular Aspiration: Development of an Experimental and Numerical Setup**

C. A. Luisi^1^; A. Amiri^1^; M. Büsen^1^; O. Nikoubashman^2^; M. Wiesmann^2^; U. Steinseifer^1^; M. Müller^2^; M. Neidlin^1^

^1^ Department of Cardiovascular Engineering, Institute of Applied Medical Engineering, Medical Faculty, RWTH Aachen University, Pauwelsstr. 20, 52074 Aachen, Germany

^2^ Clinic for Diagnostic and Interventional Neuroradiology, University Hospital RWTH Aachen, Pauwelsstr. 30, 52074 Aachen, Germany

Correspondence:

Michael Neidlin

Pauwelsstr. 20, 52074 Aachen, Germany

Tel: +49 241 80-88616

Fax: +49 241 80-82144

[neidlin@ame.rwth-aachen.de](mailto:neidlin@ame.rwth-aachen.de)

**Supplementary Methods**

Supplementary Table 1: Names of artery model segments and corresponding geometrical dimensions.

| Vessel | Mean Vessel Diameter (mm) | Vessel Centerline length (mm) |
| --- | --- | --- |
| Left internal carotid artery (LICA) | 4.9 | 163.4 |
| Right internal carotid artery (RICA) | 4.8 | 167.5 |
| Left vertebral artery (LVA) | 3.0 | 75.8 |
| Right vertebral artery (RVA) | 2.2 | 78.0 |
| Basilar artery (BA) | 3.7 | 31.8 |
| Left posterior cerebral artery (LPCA) | 2.2 | 33.1 |
| Right posterior cerebral artery (RPCA) | 1.7 | 36.1 |
| Left posterior communicating artery (LPCoA) | 1.9 | 10.1 |
| Right posterior communicating artery (RPCoA) | 2.1 | 16.2 |
| Left middle cerebral artery (LMCA) | 3.0 | 22.1 |
| Right middle cerebral artery (RMCA) | 3.2 | 20.5 |
| Left anterior cerebral artery (LACA) | 2.5 | 20.5 |
| Right anterior cerebral artery (RACA) | 2.6 | 20.0 |

Supplementary Figure 1. Cycle repeatability and flow rate assignment for LICA and RICA.

**Supplementary results**


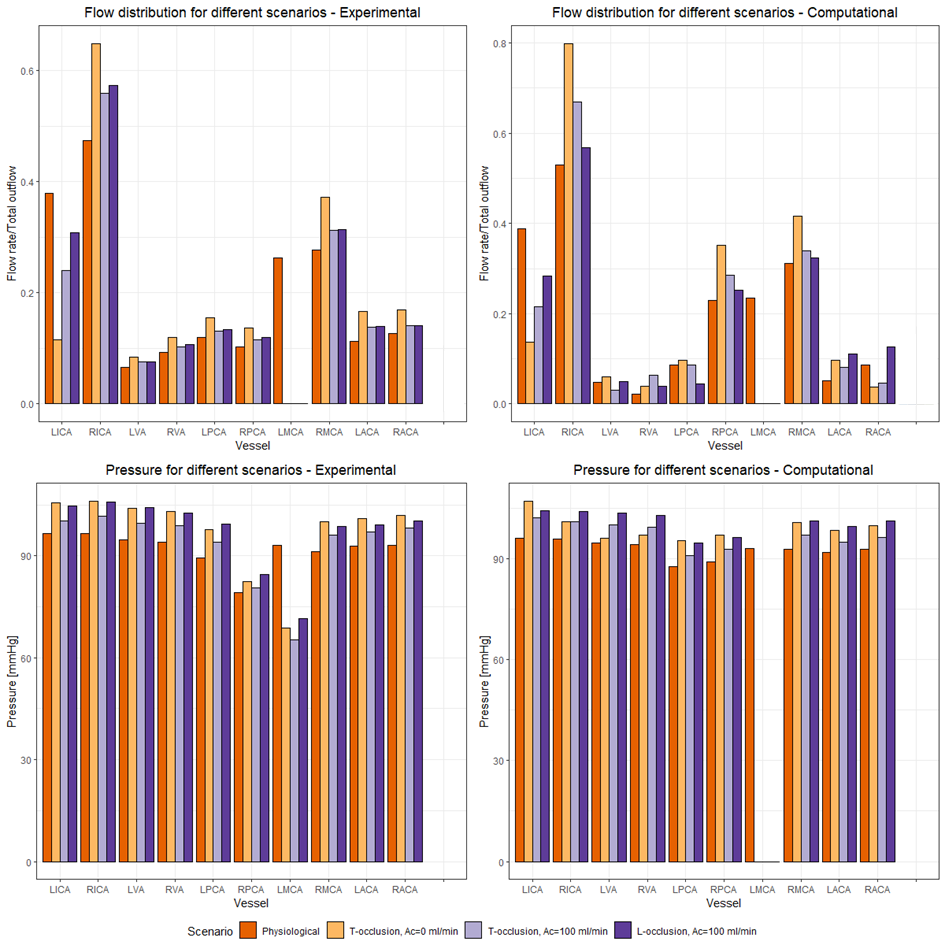


Supplementary Figure 2. Individual comparison of flows and pressures between experiment and simulation.


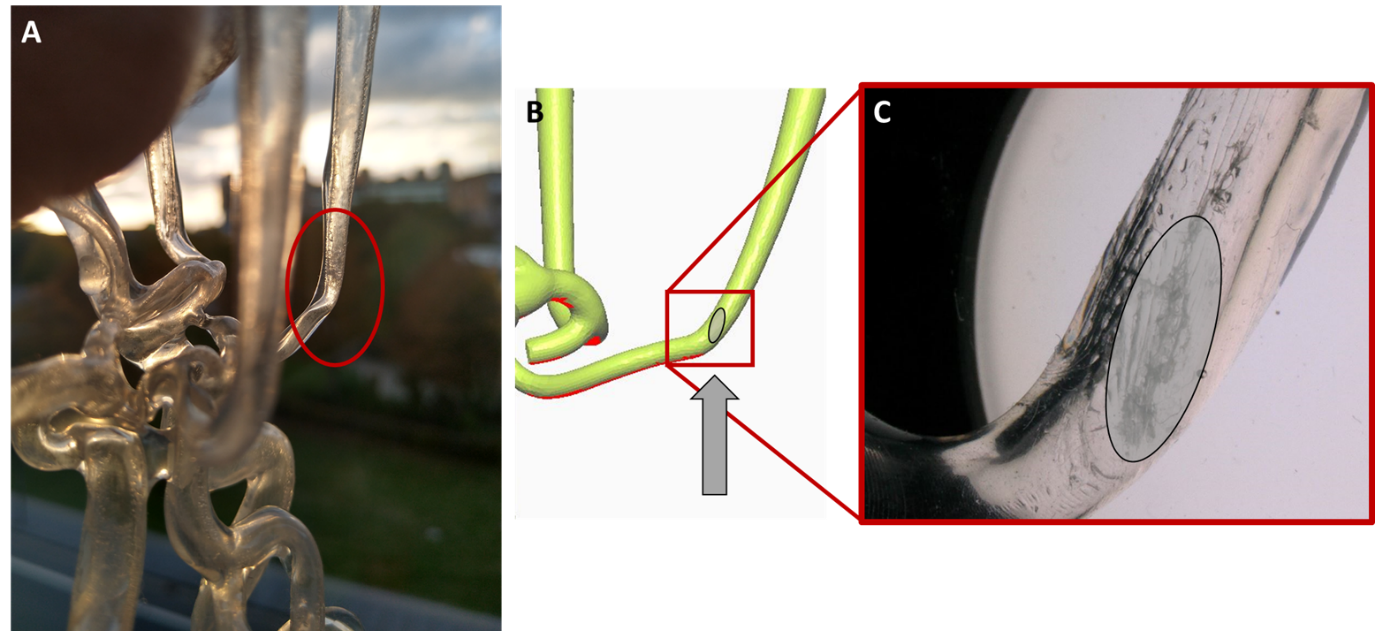


Supplementary Figure 3: Stenosis in the silicone model. A: Overall view and location in the RPCA (red circle). B: 3D model of the vessel and detailed location of the stenosis. C: Microscopic image of the stenosis as close-up for the reconstruction

Supplementary Table 2: Volume flow rates for scenario A (physiological case) for the experiment, CFD simulation without a stenosis in the RPCA and a CFD simulation with a stenosis according to Supplementary Figure 3. Bold frame: Boundary conditions.

| Vessel name | Volume flow experimental [ml/min] | Volume flow numerical  no stenosis [ml/min] | Volume flow numerical  stenosis [ml/min] |
| --- | --- | --- | --- |
| LICA | 233 | 233 | 233 |
| RICA | 291 | 291 | 291 |
| LVA | 40 | 40 | 40 |
| RVA | 57 | 57 | 57 |
|  |  |  |  |
| LPCA | 73 | 54 | 63 |
| RPCA | **62** | **144** | **101** |
| LMCA | 161 | 144 | 156 |
| RMCA | 169 | 192 | 203 |
| LACA | 68 | 32 | 36 |
| RACA | 77 | 54 | 60 |
